# S225. A SYSTEMATIC REVIEW AND META-ANALYSIS OF PHARMACOLOGICAL INTERVENTIONS FOR REDUCTION OR PREVENTION OF WEIGHT GAIN IN SCHIZOPHRENIA

**DOI:** 10.1093/schbul/sby018.1012

**Published:** 2018-04-01

**Authors:** Sri Mahavir Agarwal, Zohra Ahsan, Jonathan Lockwood, Marcus Duncan, Hiroyoshi Takeuchi, Tony Cohn, Valerie Taylor, Gary Remington, Guy Faulkner, Margaret Hahn

**Affiliations:** 1 Centre for Addiction and Mental Health; 2 University of British Columbia; 3 Women’s College Hospital

## Abstract

**Background:**

Weight gain is an extremely common problem in schizophrenia patients and is associated with morbidity and mortality. We conducted a Cochrane meta-analysis to determine the effects of pharmacological interventions aimed at reduction or prevention of weight gain in schizophrenia.

**Methods:**

We searched the Cochrane Schizophrenia Group’s Trials Register that is based on regular searches of CINAHL, BIOSIS, AMED, EMBASE, PubMed, MEDLINE, PsycINFO, and registries of clinical trials. There is no language, date, document type, or publication status limitations for inclusion of records in the register. We also searched reference sections within relevant papers, hand searched key journals, and contacted the first author of each relevant study and other experts to collect further information. We included all double blind randomized controlled trials examining any adjunctive pharmacological intervention for weight loss (treatment) or weight maintenance (prevention) in patients with schizophrenia or schizophrenia-like illnesses. We reliably selected, quality assessed and extracted data from studies. As endpoint and change data were combined in the analysis, mean differences (MD) of the change from baseline were calculated. The primary outcome measure was weight loss.

**Results:**

Forty-four randomized controlled trials met the inclusion criteria for this review. Ten studies examined prevention of weight gain while on antipsychotics. Reboxetine may be slightly effective in preventing weight gain (Weight: MD -2.09 kg, 95% CI -3.12 to -1.05; participants = 111; studies = 3; BMI: MD -0.70, 95% CI -1.03 to -0.36; participants = 111; studies = 3) and but the quality of evidence is low as the studies are small and have all been conducted by the same group. We are uncertain about the other agents used in a preventive role because the quality of evidence is very low.

Thirty-four studies examined reduction of weight gain with pharmacological interventions. Metformin is effective in bringing about modest weight loss (MD -3.45 kg, 95% CI -4.92 to -1.98 kg; participants = 569; studies = 8; BMI: MD -1.32 kg/m2, 95% CI -1.84 to -0.81 kg/m2; participants = 606; studies = 9). This effect is probably stronger in first episode psychosis (FEP) patients (Weight: MD -5.18 kg, 95% CI -6.22 to -4.14 kg; participants = 214; studies = 3; BMI: MD -1.87 kg/m2, 95% CI -2.19 to -1.56 kg/m2; participants = 214; studies = 3) compared to chronic patients (Weight: MD -2.0 kg, 95% CI -3.0 to -1.0 kg; participants = 355; studies = 5; BMI: MD -0.73 kg/m2, 95% CI -1.04 to -0.42 kg/m2; participants = 392; studies = 6). Metformin probably decreases fasting insulin levels and insulin resistance as well. The frequency of adverse effects did not differ between metformin and placebo groups. Other agents that may reduce weight include aripiprazole, topiramate, H2 antagonists such as nizatidine, and sibutramine but the quality of the evidence is low or very low making the effects uncertain. Importantly, none of the adjunctive treatment strategies result in higher dropout rates. Other than higher reports of anxiety with aripiprazole, none of the adjunctive treatment strategies appear to result in worsening of mental status.

**Discussion:**

Accumulating evidence supports the safe use of pharmacological interventions to achieve modest weight loss. Reboxetine may be slightly effective in preventing weight gain while metformin has the most evidence for use as treatment of weight gain in schizophrenia. The small number of studies, small sample size, and short study duration limits interpretation for other agents. Future studies that are adequately powered, with longer treatment duration, will be needed in evaluating the efficacy and safety of interventions for managing weight gain further.

